# Effect of a Particulate and a Putty-Like Tricalcium Phosphate-Based Bone-grafting Material on Bone Formation, Volume Stability and Osteogenic Marker Expression after Bilateral Sinus Floor Augmentation in Humans

**DOI:** 10.3390/jfb8030031

**Published:** 2017-07-29

**Authors:** Christine Knabe, Doaa Adel Khattab, Esther Kluk, Rainer Struck, Michael Stiller

**Affiliations:** 1 Department of Experimental Orofacial Medicine, Philipps University; 35039 Marburg, Germany; estherkluk@aol.com (E.K.); stiller@implant-consult.de (M.S.); 2 Department of Oral Periodontology, School of Dentistry, Ain Shams University, Cairo 11772, Egypt; doaadel12345@yahoo.com; 3 European Center of Dental Implantology (ECDI), 14193 Berlin, Germany; info@implant-consult.de

**Keywords:** tricalcium phosphate putty scaffold, bioactive ceramics, bone formation, osteogenesis, osteogenic markers, hard tissue histology, immunohistochemical analysis, split-mouth design, sinus floor augmentation, bone-grafting materials

## Abstract

This study examines the effect of a hyaluronic acid (HyAc) containing tricalcium phosphate putty scaffold material (TCP-P) and of a particulate tricalcium phosphate (TCP-G) graft on bone formation, volume stability and osteogenic marker expression in biopsies sampled 6 months after bilateral sinus floor augmentation (SFA) in 7 patients applying a split-mouth design. 10% autogenous bone chips were added to the grafting material during surgery. The grain size of the TCP granules was 700 to 1400 µm for TCP-G and 125 to 250 µm and 500 to 700 µm (ratio 1:1) for TCP-P. Biopsies were processed for immunohistochemical analysis of resin-embedded sections. Sections were stained for collagen type I (Col I), alkaline phosphatase (ALP), osteocalcin (OC) and bone sialoprotein (BSP). Furthermore, the bone area and biomaterial area fraction were determined histomorphometrically. Cone-beam CT data recorded after SFA and 6 months later were used for calculating the graft volume at these two time points. TCP-P displayed more advantageous surgical handling properties and a significantly greater bone area fraction and smaller biomaterial area fraction. This was accompanied by significantly greater expression of Col I and BSP and in osteoblasts and osteoid and a less pronounced reduction in grafting volume with TCP-P. SFA using both types of materials resulted in formation of sufficient bone volume for facilitating stable dental implant placement with all dental implants having been in function without any complications for 6 years. Since TCP-P displayed superior surgical handling properties and greater bone formation than TCP-G, without the HyAc hydrogel matrix having any adverse effect on bone formation or graft volume stability, TCP-P can be regarded as excellent grafting material for SFA in a clinical setting. The greater bone formation observed with TCP-P may be related to the difference in grain size of the TCP granules and/or the addition of the HyAc.

## 1. Introduction

Over the last 15 years, the use of resorbable synthetic ceramic bone-grafting materials has received ever-increasing attention in implant dentistry. Furthermore, augmentation of the maxillary sinus floor with autogenous bone grafts has become a well-established pre-implantology procedure for alveolar ridge augmentation of the posterior maxilla. With respect to sinus floor augmentation procedures a significant amount of bone tissue needs to be replaced in order to facilitate stable anchorage and osseointegration of dental implants in the grafted sinus floor. The success rates which have been achieved when using calcium phosphate-based bone substitute materials for sinus floor augmentation (SFA) demonstrate that these materials have become an excellent alternative graft choice compared to autogenous bone grafts, which have commonly been considered as the gold standard [1,2,3]. In addition, using biodegradable bone substitutes does simplify sinus floor elevation procedures, since it avoids second-site surgery for autograft harvesting thereby eliminating the risk of donor-site morbidity [4,5,6]. Among the bone substitutes materials available, β-tricalcium phosphate (β-TCP) ceramic has achieved widespread use for SFA procedures [6,7,8,9,10,11,12]. The macro-, meso- and micro-porosity, particle size and shape, geometry at the macro- and micro scale, mechanical properties, phase purity, solubility, and application form, i.e., blocks, granules, TCP-based cements or putties, as well as the presence of polymeric and other supplements—in the case of these TCP-based cements and putties—affect the osteogenic potential and degradation properties of these TCP-based biomaterials [2,7,13,14,15].

More recently, the combination of tricalcium phosphate ceramics with polymeric scaffolds or carriers including natural polymers has received increasing attention in scientific research [7,13]. Among the candidate biomaterials for this type of application hyaluronic acid has shown to be clinically advantageous due to its excellent biologic properties [7,16]. Hyaluronic acid (HyAc) as well as its polyanionic form hyaluronate is a highly molecular polysaccharide, which is a major component of the extracellular matrix of the skin, tendons, muscles, articular cartilage and the synovial fluid of vertebrates. Moreover, HyAc plays an important role in the body’s osmoregulation due to its interaction with binding proteins, proteoglycans and other bioactive molecules. In addition to being immunologically inert HyAc also has a stimulatory effect on angiogenesis [17]. Due to these advantageous properties there has been an ever-increasing use of HyAc in various medical disciplines such as regenerative medicine, traumatology, rheumatology, and cosmetic medicine [16,18,19,20,21]. Furthermore, these physicochemical and biological properties render HyAc an attractive material for combining it with β-TCP granules with the intent to optimize the regenerative potential of β-TCP and to create a putty-like bone-grafting scaffold material with improved surgical handling properties for bone reconstruction of defects with demanding defect morphologies. SFA procedures fall into this category. SFA aims at creating sufficient bone volume in the atrophic posterior maxilla in order to facilitate stable and reliable anchorage of dental implants in cases of a severely reduced residual bone height of less than 3 mm of the residual partially edentulous alveolar ridge. While other bony defects in the alveolar ridge such as lateral bone defects often are three- or five-wall defects, which can easily be accessed surgically, a more difficult situation and greater challenge is faced in the case of SFAs. There is only one bony wall present, i.e., the sinus floor. The bone-grafting material needs to be introduced via the lateral surgically created access window to the sinus and has to be carefully placed underneath the elevated Schneiderian membrane with its delicate and vulnerable structure [22,23]. Thus, special care needs to be taken in order to ensure that during the grafting process the Schneiderian membrane remains intact without any perforations, since the success of SFA procedures is severely challenged, if the Schneiderian membrane is damaged during the surgical procedure [24,25]. Consequently, from a clinical point of view, it is of great importance to investigate the use of TCP-based bone-grafting materials different from granules such as putty-like scaffold materials for SFA and to compare these putty-like TCP-based materials to TCP-granules with the aim to improve surgical handling and reduce surgical complications. The clinical success rates, which are achieved when using TCP granules for SFA, have been widely documented in the scientific literature [2,6,7,8,9,10,11,12].

In the present clinical study a randomized split-mouth study design was used to evaluate the effect of two β-TCP-based bone substitute materials, i.e., a TCP putty scaffold composed of TCP granules embedded in a fermented sodium hyaluronate hydrogel carrier and TCP granules of equal chemical composition but different grain-size, on bone formation and osteoblast differentiation after SFA in humans. This also included assessing, whether the volume of the augmented bone was maintained over time using cone-beam CTs. Hence, the objective of this study was to elucidate, whether by combining TCP granules with a fermented sodium hyaluronate carrier surgical handling properties, bone formation and stability of the augmented bone volume can be enhanced, which constituted the hypothesis to be tested.

## 2. Materials und Methods

### 2.1. Test Materials

Test materials were two commercially available β-TCP-based bone-grafting materials. First pure, synthetic β-TCP granules with a porosity of 60% and a grain size of 700 to 1400 µm (material denominated TCP-G; CEROS^®^ TCP Granules, MathysMedical, Bettlach, Switzerland). This material (with the commercial name CEROS, when used for carnioimaxillofacial applications and named CHRONOS, when used for orthopedic application) has been used since 1982, with a considerable body of knowledge having been gathered in the scientific literature since, and its fabrication process and material properties having been characterized in detail in previous publications [26,27,28,29,30,31,32,33,34,35,36]. In summary, TCP-G granules display interconnected macropores (100–500 µm in size) in combination with a fraction of micropores (1–10 μm). The detailed pore size distribution is as follows (pores < 100 µm: 0.3%; pores 100–160 µm in size: 12.9%; pores 160–240 µm in size: 33.6%; pores 240–320 µm in size: 29.5%; pores 320–400 µm in size: 16.6%; pores 400–500 µm in size: 6.9%; pores > 500 µm in size: 0.3% [29]. The phase purity of the β-TCP has been confirmed previously by Walsh et al. (2008) [36]. The second test material was a putty-like β-TCP-based scaffold composed of pure β-TCP granules of identical chemical composition, with a porosity of 60% and two types of grain size (125–250 µm and 500 to 700 µm, combined at a 1:1 ratio) embedded in a fermented sodium HyAc hydrogel matrix (material denominated TCP-P; CEROS^®^ TCP Putty, MathysMedical, Bettlach, Switzerland). Characterization of the material properties was performed previously by Bohner et al. (2010) [7]. The TCP granules for both materials are fabricated using the same manufacturing process. TCP blocks with the interconnected porosity described above are created by coating a polymer template with the ceramic TCP slurry and subsequently sintered at 1000 °C–1300 °C [30], then crushed, ground and sieved to obtain granules with different grain sizes of 700 to 1400 µm (TCP-G), 125 to 250 µm and 500 to 700 µm (TCP-P). The density of the β-TCP ceramic itself is 3.1 g/cm^3^ [7]. The packing density (volume taken by a given weight of granules when placed in a graduated cylinder) is 0.6–0.65 g/cm^3^ for the 700 to 1400 µm and 500 to 700 µm grain size fractions, and 0.9 g/cm^3^ for the 125 to 250 micrometer grain size fractions. The TCP-P has two components, which are mixed by the surgeon in the operating room. Component I consists of the pure β-TCP granules (94%) [7] described above. Component II is composed of 6% recombinant sodium hyaluronate powder, which is mixed with physiological saline or venous blood in the operating room, and then mixed with the granules at a ratio 1:10 prior to prior to delivery into the surgical site [7]. Also TCP-G was mixed with venous blood to achieve a paste like consistency for surgical handling. Finally, autogenous bone chips harvested from the tuber maxillae were added to both TCP-G and TCP-P prior to delivery into the sinus cavity at a ratio of 1:10. TCP-G and TCP-P were kindly provided by the RMS Foundation.

### 2.2. Patient Selection

The study consisted of 7 patients (5 women and 2 men) ranging in age from 57 to 72 years (mean 65.4 years). The patients chosen were partially edentulous in the post-canine region bilaterally. In all patients bilateral SFA was required in order to facilitate dental implant placement in the posterior maxilla with the height of the residual alveolar crest being less than 3 mm. A split-mouth design was used in the current study. After routine oral and physical examinations the patients were selected and SFA procedures were planned. The patients’ data are listed in Table 1. Patients were excluded from the study, if their health was compromised (ASA (III or IV)—American Society of Anaesthesiology) and if they were suffering from any drug abuse, including alcohol, or any significant systemic disease. All patients had good oral health and no active periodontitis. Patients with radiological signs of pathological changes of the maxillary sinuses were excluded. 5 of the patients selected were non-smokers two patients had a history of smoking 5 cigarettes daily and were instructed to stop smoking at least 2 weeks prior to the first surgery and for 40 weeks after the second surgery. These two patients were informed of the increased failure risk due to smoking. In all patients the width of the alveolar crest was greater than 6 mm so as to facilitate sampling biopsies in a safe and easy manner. The Freiburg Ethics Commission International approved the study. All participants were fully informed about the procedures, including the surgery, bone substitute materials and implants, and provided written informed consent (registry number DRKS00007538).

### 2.3. Radiological Examination

#### 2.3.1. Cone Beam CT

Cone-beam computed tomography (CT) (KaVo-3D- eXam^®^, KaVo Dental GmbH, Germany) was used in all patients for 3D assessment of the sinus floor anatomy and bone volume preoperatively, postoperatively and 6 months after SFA. The cone-beam CTs of the maxilla were acquired with a voxel size of 0.25 mm and an exposure time of 26 s. Cone-beam CT facilitates three-dimensional visualization of the maxillary sinuses with a limited radiation exposure. The first preoperative cone beam CT, i.e., primary cone-beam CT, was obtained in order to evaluate the residual bone volume of the alveolar ridge, to exclude pathological changes of the maxillary sinuses and for treatment planning. The secondary, i.e., postoperative cone-beam CT, documented the outcome of the grafting procedure and checked for possible entrapment of air bubbles within the grafting material, possible dislocation of the grafting material and possible hemorrhages and mucus retention in the maxillary sinus. The third cone beam CT, i.e., the tertiary cone-beam CT, was obtained directly after implant placement and biopsy sampling 6 months after SFA in order to determine the volume of the grafted bone and to document the outcome of the implant placement surgery. Consequently, the cone-beam CT-data allowed following the changes in volume of the grafted area occurring during the six-month healing period.

#### 2.3.2. Data Transfer, Analysis and Volume Determination

**Figure 1:**
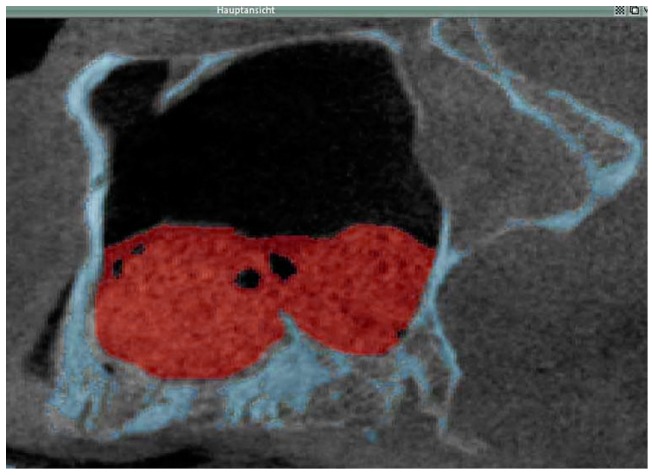
Two-dimensional radiographic image generated from the secondary cone beam CT acquired directly after SFA showing of a cross section through the augmented sinus floor (red) as well as the osseous anatomical structures of the native sinus floor (blue). This image was generated by merging the data sets of the primary and secondary cone-beam CTs. Sagittal view of the, left-sided maxillary sinus, patient 5.

**Figure 2:**
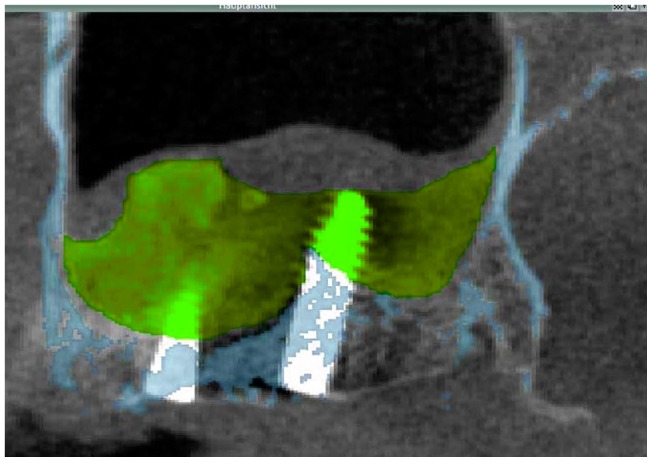
Two-dimensional radiographic image generated from the tertiary cone-beam CT acquired at implant placement 6 months after SFA showing of a cross section through grafted area after the 6 months healing period (green) as well as the osseous anatomical structures of the native sinus floor (blue). This image was generated by merging the data sets of the primary and tertiary cone-beam CTs in combination with superimposing the image of the secondary cone beam CT (light grey). Sagittal view of the left-sided maxillary sinus, patient 5.

For each patient the DICOM (Digital Imaging and Communications in Medicine) data sets of the three cone-beam CTs acquired were imported into a three-dimensional image processing software (VoXim^®^, IVS Technology GmbH, Chemnitz, Germany), which allowed to determine the volume of the grafted areas after SFA. To this end, a segmentation of the volume data of the grafted sinus with subsequent visualization was performed. This way, the volume of the grafted area obtained by the SFA procedure was compared to that present after 6 months of graft healing. For the transfer of the DICOM data sets, the gray value distribution and their limits were set equivalent to the Hounsfield scale that is commonly used in regular spiral computed tomography (CT), i.e., level = 665, width = 3379 and Hounsfield units = 1024 to 2354. Between 240 and 448 slices for each data set, i.e., each maxillary sinus, were analyzed. The regions of interest (ROIs) of the augmented area were manually selected for each data set. The volume was automatically calculated by the software by calculating the sum of the areas formed by the manually marked regions. The implants were included in the ROIs for the set of the tertiary cone-beam CTs; this was done in order to determine to the bone volume present just prior to implant insertion. Since during data transfer the VoXim^®^ software (version 5.6) sets the pixel size at 0.31 mm and the interlayer distance at 0.2 mm, in the primary cone-beam CT a threshold value segmentation for the bony structures was performed in order to mark and visualize the bony margins of the maxillary sinus prior to surgery. This was followed by merging the data sets of the primary and secondary cone beam CTs. This way it was possible when segmenting the values for the grafted area in the secondary cone-beam CT to then also visualize the bony margins of the maxillary sinus prior to surgery (Figure 1). Segmenting the data of the grafted area after SFA facilitated visualizing and measuring the volume of the grafted area in mL. The same steps were followed for calculating the volume of the augmented area 6 months after SFA at implant placement (Figure 2). The anatomical structures of interest such as the grafted area and surrounding bony structures were highlighted using different colors. In order to visualize the decrease in volume of the grafted area between the time point of SFA and the time point of implant placement 6 months later the images of the secondary and tertiary cone beam CT were superimposed for each patient (Figure 2).

### 2.4. Sinus Floor Elevation

Bilateral sinus floor elevation was performed under local anesthesia by the same experienced surgeon in each of the seven patients. A staged surgical approach was used, as the height of the residual alveolar process was <3 mm in the posterior maxillary region. The space created between the maxillary alveolar process and the elevated Schneiderian membrane was filled using a combination (10:1 ratio) of β-TCP granules (TCP-G) or β-TCP-putty (TCP-P) and autogenous bone chips. Bone chips milled into very small particles and added to the biomaterial.β-TCP granules (TCP-G) were implanted in either the left- or right-sided maxillary sinus floor according to a randomized protocol, while when augmenting the contralateral sinus floor the TCP putty was used. Small amounts of autogenous bone chips were harvested in all patients from the tuber maxillae. The TCP-G and TCP-P grafting material was mixed with venous blood prior to delivery into the open sinus cavity. In order to prevent infections, all patients received 1200 mg of clindamycin (Clindamycin ratiopharm 600 mg, Ratiopharm GmbH & Co., Ulm, Germany) daily for 7 days. This was in addition to an intravenous injection of 250 mg prednisolone (Solu-Decortin H 250, Merck KGaA, Darmstadt, Germany) in combination with daily oral administration of 800–1200 mg of ibuprofen (IBU ratiopharm 400 akut, Ratiopharm GmbH & Co., Ulm, Germany) to reduce pain and swelling. Furthermore, a local submucosal infiltration of 1 mL dexamethasone was administered in the vestibular region bilaterally (Dexabene 4 mg/mL, Merckle-Recordati KGaA, Darmstadt, Germany) to reduce postoperative pressure formation on the graft resulting from swelling of the Schneiderian membrane.

### 2.5. Dental Implant Surgery and Biopsy Specimen Retrieval

After 6 months of healing the patients received implants. Dental implant placement and bone biopsy sampling were carried out under local anesthesia. In each patient one biopsy was harvested from each site, in which a dental implant was to be placed, using a trephine burr (3.5 mm outer diameter and 2.75 mm inner diameter, reference number 044.328, Straumann^®^, Basel, Schweiz) with copious saline irrigation. The site, in which the height of the original residual alveolar process (prior to augmentation) was approximately 1–3 mm, was chosen for biopsy. The biopsies sampled from all patients were 2.5 mm in diameter and approximately 8 mm in length depending on the mechanical stability of the regenerated tissue. These specimens were used for histomorphometric and immunohistochemical evaluation. The specimens contained both the grafted area and the residual native crest, which was approximately 1–3 mm in height. This residual native crest was not included in the histomorphometric analysis.

### 2.6. Preparation of Biopsy Specimens for Histomorphometry and Immunohistochemistry

The bone specimens were processed using a technique that facilitated performing immunohistochemical analysis on undecalcified hard tissue sections as previously described [2,37,38]. The tissue samples were immediately fixed in an ethanol-based fixative HistoCHOICE^R^ (AMRESCO, Solon, OH, USA.) at room temperature (20–22 °C) for 5 days. This was followed by dehydratation and infiltration. Subsequently, the specimens were embedded in a resin, which was composed of pure metylmetacrylate and n-butyl-metacrylate to which benzoyl peroxide (BPO catalyst, Merck KGaA, Darmstadt, Germany) and polyethylene glycol 400 (Merck KGaA, Darmstadt, Germany) and 1.5 mL N,N-dimethyl-p-toluidine (Merck KGaA, Darmstadt, Germany) were added. Samples were polymerized in polyethylene vials at 4 °C for 4–7 days. This resin was selected because it maintained the antigenicity of the tissue and at the same time also provided adequate material properties for cutting 50 µm thick sections with a sawing microtome. After polymerization the blocks were removed from the vials and excess resin was trimmed away. These blocks were glued to acrylic slides (Plexiglas GS209, Röhm, Arnsberg, Germany) using an epoxy resin based two-component adhesive (UHU, Bühl, Germany). Sections (50 μm) were cut using a Leitz 1600 sawing microtome (Leitz, Wetzlar, Germany) and then ground and polished. Subsequent to deacrylation of the sections by immersion in toluene, xylene and acetone, immunohistochemical staining was performed using primary mouse monoclonal antibodies specific to alkaline phosphatase (ALP) (Sigma-Aldrich, Steinheim, Germany), osteocalcin (OC) (Abcam, Cambridge, UK), and rabbit polyclonal antibodies against type I collagen (Col I) (LF-39, NIH, Bethesda, Rockville, MD, USA) and bone sialoprotein (BSP) (LF-84, NIH, Bethesda, MD, USA) [2,37,38,39]. Other sections were stained for Tartrate Resistant Acid Phosphatase (TRAP) activity to identify cells with osteoclastic activity using a prefabricated kit (Sigma-Aldrich, Steinheim, Germany) which is based on the method described by Goldberg and Barka (1962) [40]. This procedure stains cells with TRAP activity. Mayer’s hematoxylin was used as a counterstain. Sections obtained from experimental fracture healing sites in male Wistar rats were used as positive controls, since osteoclasts were known to be present. Non-immunized mouse, and rabbit IgG (PP54 and PP64) (Millipore, Billerica, MA, USA) were applied as negative controls. The latter ruled out the non-specific reactions of mouse and rabbit IgG to human tissues, as well as non-specific binding of the secondary antibodies and/ or peroxidase labeled polymer to human tissues. Incubation with a peroxidase labeled dextran polymer conjugated to goat anti-mouse and anti-rabbit immunoglobulins (DAKO DakoCytomation Envision+ Dual link system peroxidase, DAKO, Glostrup, Denmark) followed and the color was developed using a liquid 3-amino-9-ethylcarbazole (AEC) system (DAKO, Glostrup, Denmark), as described previously [2,37,38]. Mayer’s hematoxylin was used as a counterstain, and Kaiser’s glycerol gelatin (Merck KGaA, Darmstadt, Germany) was utilized for mounting with coverslips. 3-amino-9-ethylcarbazole results in a bright red and/or slightly brownish color, while counterstaining with hematoxylin yields a violet/bluish color. As a result, positively stained cells and matrix components can be distinguished from cells and matrix components with negative immunostaining at the appropriate magnification [2,37,38]. Histomorphometric analysis was performed on a pair of sections 150 μm apart. The sections were measured semiautomatically using a light microscope (Vanox-T AH2, Olympus, Hamburg, Germany) in combination with a digital camera (Colorview IIIu, Olympus, Hamburg, Deutschland) and Analysis™ software (Olympus, Hamburg, Germany). To this end, a rectangular area 6 mm^2^ in size (3 mm long and 2 mm wide) was defined (at a distance of 3 mm from the native alveolar crest and extending in an apical direction) in in each section as ROI (region of interest) [38]. In each ROI the surface area that consisted of newly formed bone, and the area that consisted of graft material was measured in mm^2^ and the area fraction of newly formed bone (bony trabeculae) as well as the area fraction of grafted material (denominated biomaterial or particle area fraction) was analyzed as percentage of the total. Data from each pair of section were averaged.

Furthermore, semi-quantitative analysis of the immunohistochemically stained sections was performed as described previously [2,38,41,42]. The stained sections were analyzed under the light microscope by two experienced investigators with both investigators blinded to the staining. For the immunohistochemical evaluation the rectangular area 6 mm^2^ in size used for the histomorphometric evaluation was divided in a central and apical area of equal size, i.e., 3 mm^2^ each. The tissues were examined for antibody decoration of cellular and matrix components. The cellular components examined included fibroblasts, osteoblasts, osteocytes and osteoclasts. The matrix components included trabecular bone, osteoid seams, bone marrow spaces and fibrous matrices. All these histological components were identified on morphological grounds. A scoring system quantified the amount of staining observed using light microscopy [2,38]. A score of (**+++**[=5]), (**++**[=4]), and (**+**[=2]) corresponded to generalized strong, moderate or mild staining, whereas a score of (+++[=4]), (++[=3]), and (+[=1]) corresponded to strong, moderate or mild staining in localized areas. A score of (0) correlated with no staining. The scores for the amount of staining of a given cellular or matrix component for a respective marker were averaged for both the apical and central areas. This also included subjecting the individual scores to statistical analysis. An average score of 3.5–5 was evaluated as strong expression in the given grafting group, whereas an average score of (2.3–3.4), (1–2.2), and (0.1–0.9) was assessed as moderate, mild and minimal expression [2,38].

### 2.7. Statistical Analysis

The student’s paired *t*-test was used to determine statistical significance (SPSS software, version 21). Values of *p* < 0.05 were considered to be significant.

## 3. Results

### 3.1. Clinical Findings

After SFA, no postoperative complications occurred in any of the patients. Normal wound healing was observed both after SFA and implant placement surgeries. The TCP-P material displayed more advantageous surgical handling properties, since it facilitated introducing the grafting material into the sinus floor as well as condensing it in a more easily. Six months after SFA all patients had sufficient bone levels for placement of the implants with adequate primary stability. A total of 40 dental implants were inserted into the augmented maxillary sinus floors of 7 patients. Intraoperatively, no differences were noted with respect to drilling resistance when preparing the implant bed in sites, in which TCP-P was used compared to sites augmented with TCP-G. Cone-beam CTs did not reveal any pathological changes in the augmented sinuses or the surrounding tissues. There was no incidence of sinusitis or perforations of the Schneiderian membrane in any of the patients. Biopsies varied in length between patients No implant failures were noted up to the time of the completion of this manuscript (6 years after implant placement; details s. Section 3.4).

### 3.2. Radiological Results

**Figure 3:**
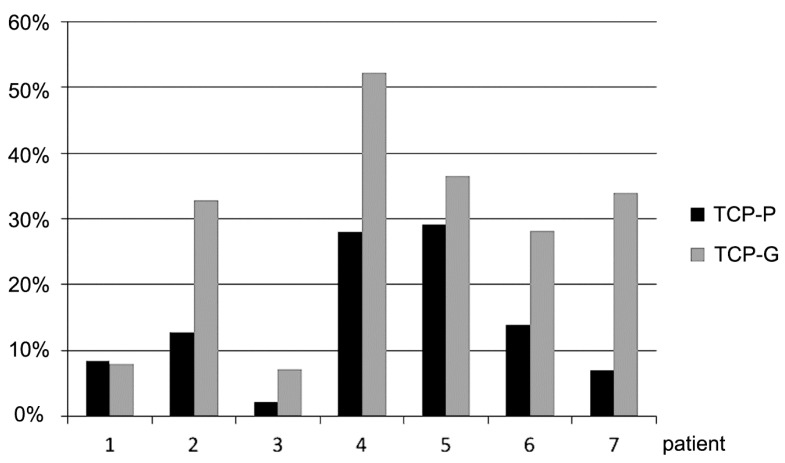
Decrease (%) in grafting volume observed 6 months after sinus floor augmentation with TCP-putty and TCP granules in 7 individual patients.

**Figure 4:**
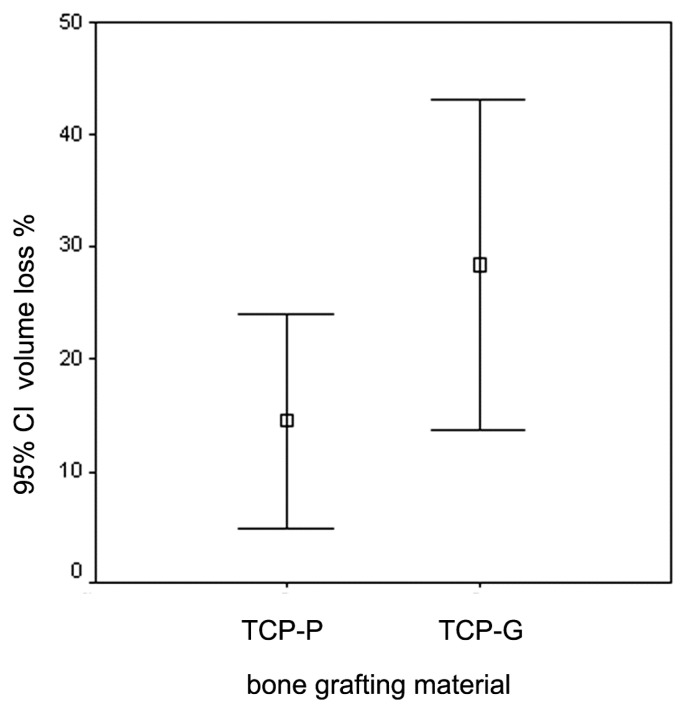
Graph depicting the mean values and 95% confidence interval (CI) for the decrease in grafting volume 6 months after SFA using CEROS^®^-TCP-putty (TCP-P) and CEROS^®^-TCP-granules (TCP-G).

Analysis of the cone-beam CT data revealed a decrease in volume of the grafted area in the sinus floor in all cases 6 months after SFA (Figure 3 and Figure 4). This reduction in volume was visible in in the periphery of the grafted area with shrinkage towards the center. Sites that were grafted with TCP-G showed a greater reduction in volume 6 months after SFA compared to sites in which TCP-P was used. Figure 3 shows the reduction in volume of the grafted area during the 6 months healing period for each patient.

In all patients, except for patient no. 1, a markedly greater reduction in graft volume was noted in the sites, in which TCP-G was used compared to sites that were grafted with TCP-P. In patient No. 1 the reduction in graft volume for TCP-G was only slighter greater compared to TCP-P. The mean values for the reduction in graft volume for TCP-G (28.4 ± 16.1%) were higher compared to the putty group (14.5 ± 10.3%). However, these differences were not statistically significant (*p* = 0.39, *t*-test). It is also noteworthy that secondary cone-beam CTs taken directly after sinus floor augmentation revealed entrapment of air in 4 of the 7 sites in which TCP-G was used, while this was only the case in one out of 7 sites, in which TCP-P was used. However, at implant placement, i.e., 6 months after SFA (tertiary cone-beam CT), entrapment of air was not observed in any of the patients. With respect to the Schneiderian membrane in 9 of the 14 grafted sinus floors, cone-beam CTs recorded 6 months after SFA did not show any changes of the Schneiderian membrane, while in the remaining 5 sites, three of which were grafted with TCP-P and two of which were grafted with TCP-G, a slight swelling of the Schneiderian membrane was present.

### 3.3. Results of Histologic, Histomorphometric and Immunohistochemical Analyses

**Figure 5:**
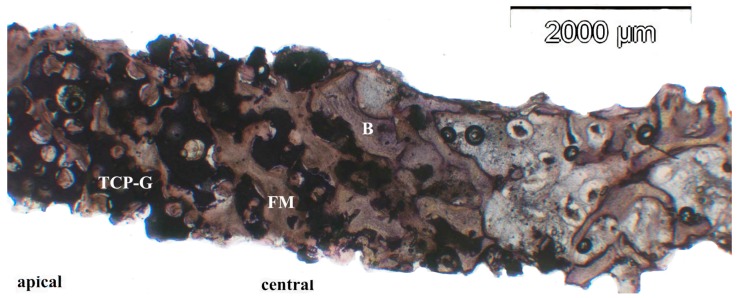
Histomicrograph of resin embedded biopsy stained immunohistochemically for osteocalcin after deacrylation. The biopsy was sampled 6 months after augmentation of the sinus floor with TCP-G (B = bone, FM = fibrous matrix of the osteogenic mesenchym, TCP-G—residual TCP particles displaying a scalloped morphology). Undecalcified sawed section counterstained with hematoxylin. Bar = 2000 µm.

**Figure 6:**
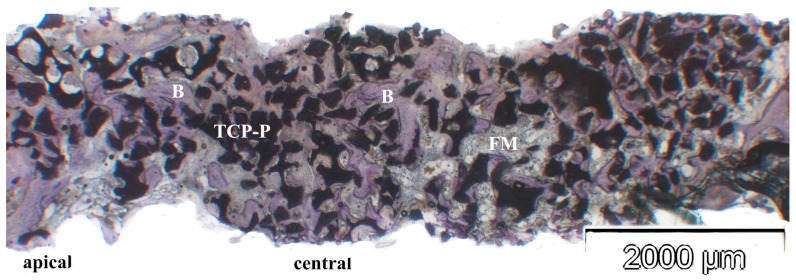
Histomicrograph of resin embedded biopsy stained immunohistochemically for osteocalcin after deacrylation. The biopsy was sampled 6 months after SFA with TCP-P (B = bone, FM = fibrous matrix, TCP-P = residual TCP particles of the putty scaffold material. The smaller grain size of these particles compared to the TCP-G material is evident. Undecalcified sawed section counterstained with hematoxylin. Bar = 2000 µm.

**Figure 7:**
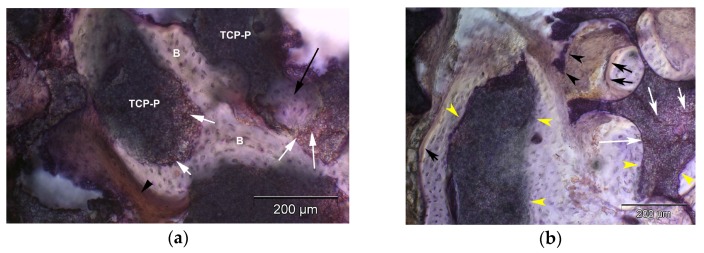

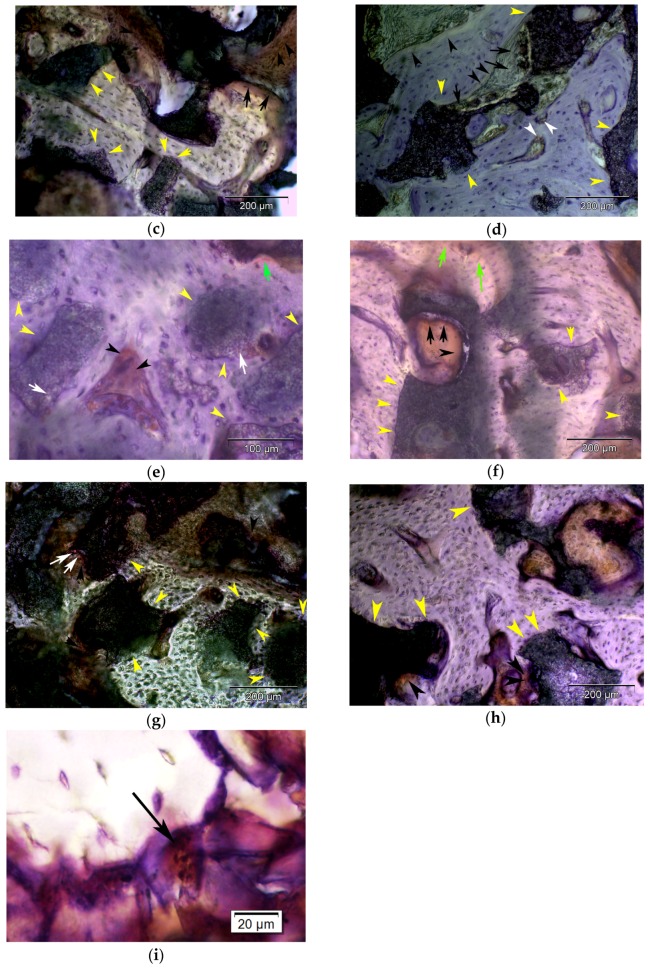
Histomicrographs of resin embedded biopsies sampled 6 months after SFA with TCP-P or TCP-G stained immunohistochemically for the osteogenic markers bone sialoprotein (**a**,**b**), osteocalcin (**c**,**d**), type I collagen (**e**,**f**), alkaline phosphatase (**g**,**h**) after deacrylation: (**a**) Immunodetection of bone sialoprotein in sawed section of biopsy sampled 6 months after SFA floor with TCP-P. Intense staining of osteoblasts, which have migrated into the degrading TCP-P particles is visible (white arrows), which exhibit excellent bone (B)-particle contact, i.e., bone-bonding behavior. Furthermore, bone formation within the degrading particles is visible (black arrow) as well as strong staining of the mineralizing but not yet fully mineralized bone matrix (black arrowhead) in contact with the TCP-P particles; (**b**) Immunodetection of bone sialoprotein in sawed section of biopsy sampled 6 months after SFA with TCP-G. Intense staining of osteoblasts, which have migrated into the degrading TCP-G particles is visible (white arrows), which exhibit excellent bone particle contact, i.e., bone-bonding behavior (yellow arrowheads). Furthermore, mild staining of the osteoid (black arrows) and mineralizing but not yet fully mineralized bone matrix, i.e., osteogenic mesenchym, (black arrowheads) in contact with the TCP-G particles is present; (**c**) Immunodetection of osteocalcin in hard tissue section of biopsy sampled 6 months after SFA floor with TCP-P. TCP-P-particles are visible, which exhibit excellent bone particle contact, (yellow arrowheads). Furthermore, strong staining of the osteoid (black arrows) and mineralizing but not yet fully mineralized bone matrix (black arrowheads) in contact with the TCP-P particles is present; (**d**) Immunodetection of osteocalcin in section of biopsy sampled 6 months after SFA floor with TCP-G. TCP-G-particles are present, which exhibit partial bone particle contact (yellow arrowheads). Osteoid (black arrows) and mineralizing but not yet fully mineralized bone matrix (black arrowheads) without any positive staining for osteocalcin are visible. In addition, osteoid with mild osteocalcin expression (white arrowheads) lining marrow spaces is visible. Undecalcified sawed section counterstained with hematoxylin. Bar = 200 µm; (**e**) Immunodetection of type I collagen in section of biopsy sampled 6 months after SFA floor with TCP-P. Intense staining of osteoblasts, which have migrated into the degrading TCP-P particles is visible (white arrows), which exhibit excellent bone-particle contact (yellow arrowheads). Furthermore, strong staining of the mineralizing but not yet fully mineralized bone matrix (black arrowheads) as well as of the fully mineralized bone matrix (green arrow) in contact with the TCP-P particles is present. Bar = 100 µm; (**f**) Immunodetection of type I collagen in section of biopsy sampled form TCP-G site. TCP-G particles are present, which exhibit partial bone particle contact (yellow arrowheads). Furthermore, moderate staining of the osteoid (black arrows) and mineralizing but not yet fully mineralized bone matrix (black arrowhead) in contact with the TCP-G particles is visible. This is in addition to mild staining of the mineralized matrix (green arrows); (**g**) Immunodetection of alkaline phosphatase in section of biopsy sampled from TCP-P site. TCP-P particles are visible, which exhibit excellent bone particle contact (yellow arrowheads). Furthermore, strong staining of the osteoblasts (white arrows) and of the mineralizing but not yet fully mineralized bone matrix (black arrowheads) in contact with the TCP-P particles is present; (**h**) Immunodetection of alkaline phosphatase in sawed section of biopsy sampled 6 months after SFA floor with TCP-G. TCP-G particles are present, which exhibit excellent bone particle contact (yellow arrowheads). Strong staining of the mineralizing but not yet fully mineralized bone matrix (black arrowheads) in contact with the TCP-G particles is visible. Bar = 200 µm; (**i**) histomicrograph of positive control section of experimental fracture site stained for TRAP activity enzymhistochemically. A TRAP-positive multinucleated osteoclast (black arrow) is visible. Bar = 20µm.

In the biopsies from sites, in which TCP-G or TCP-P was used, the β-TCP graft material was present as achromatic scalloped granules, depending on the phase of resorption (Figure 5, Figure 6 and Figure 7). The size of these residual particles, however, was significantly larger for TCP-G (Figure 5) compared to TCP-P (Figure 6), i.e., reflected the difference in grain-size used for the native TCP-G material compared to the putty. Newly formed bone was visible at the surface of these residual particles (Figure 5, Figure 6 and Figure 7). Six months after SFA neither with TCP-G nor with TCP-P complete resorption of the grafting material had occurred (Figure 5, Figure 6 and Figure 7). Resorption of the grafting materials appeared to be due to chemical dissolution rather than osteoclastic activity, since TRAP positive osteoclasts were neither observed with TCP-G nor TCP-P, while in the positive controls (experimental fracture healing sites) multinucleate TRAP positive cells were present (Figure 7i). The residual granules were partially embedded in newly formed bone, which was predominantly lamellar bone without any signs of inflammatory tissue response in any of the grafted sites (Figure 5, Figure 6 and Figure 7). With both TCP materials bone formation was preceded by the abundant proliferation of a cell rich-osteogenic mesenchyme with positive expression of Col I, BSP and OC in cell and matrix components indicating progressing matrix mineralization (Figure 5, Figure 6 and Figure 7, Table 2). Good bone bonding behavior was observed with both materials as well as bone formation within the degrading particles, which was more advanced in the TCP-P group (Figure 6 and Figure 7). This was accompanied by expression of Col I, ALP, BSP and OC in osteoblasts of the newly formed bone in contact with both types of TCP particles (Figure 5, Figure 6 and Figure 7) as well as in the fully mineralized bone matrix and osteoid (Figure 7a–h). Bone formation was more abundant in the central area compared to the apical area.

**Figure 8:**
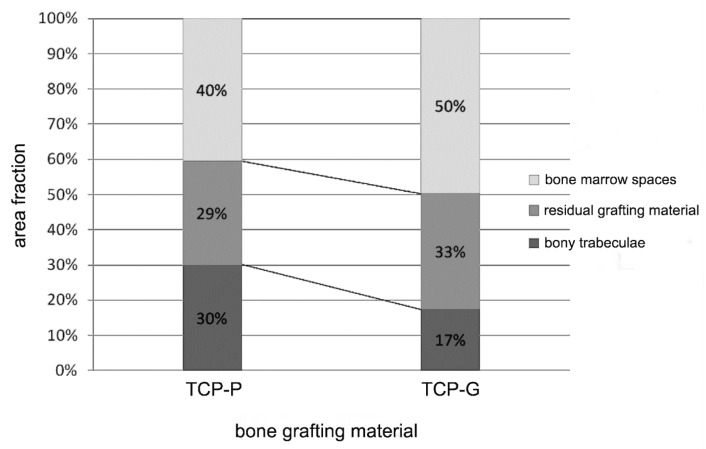
Histogram illustrating the results of the histomorphometric evaluation (mean values) of the area fraction of the newly formed bony trabeculae, of the biomaterial/particle area fraction and the area fraction of the bone marrow spaces in biopsies sampled bilaterally from seven patients 6 months after SFA with TCP-P and TCP-G.

**Figure 9:**
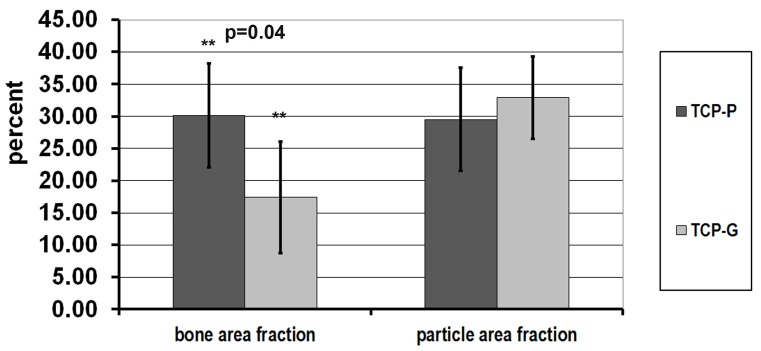
Histogram depicting the results of the histomorphometric analysis (mean values ± SEM) of the area fraction of the newly formed bony trabeculae and of the particle (residual biomaterial) area fraction in biopsies obtained bilaterally from seven patients 6 months after SFA with TCP-P and TCP-G. Asterisks indicate statistical significance.

Figure 8 and Figure 9 show the results of the histomorphometric assessment. In the grafted sinus floors, in which TCP-P was used, the mean bone area fraction (excluding marrow spaces) was 30.1 ± 8.1%, and the mean particle (biomaterial) area fraction was 29.5 ± 8.0%, whereas in the sites, in which TCP-G was used, a mean bone area fraction of 17.4 ± 8.7%, a mean biomaterial area fraction of 32.9 ± 6.4% were noted (Figure 8 and Figure 9). Thus, in the TCP-P group more bone had formed in the grafted sinus floors compared to the TCP-G group with the difference being statistically significant (*p* = 0.04). This was associated with a slightly smaller amount of residual TCP grafting material being present in the TCP-P group after 6 months of implantation (Figure 8 and Figure 9). However, this difference was not statistically significant (*p* = 0.41).

Table 2 summarizes the results of the immunohistochemical analysis. In biopsies harvested from sites grafted with TCP-P, both apically as well as centrally significantly higher expression of BSP (*p* = 0.03, apically; *p* = 0.02 centrally) was noted in osteoblasts and significantly greater Col I expression in the osteoid (*p* = 0.03) when compared to TCP-G sites (Table 2). This was in addition to significantly greater Col I expression in osteoblasts apically (*p* = 0.03). Furthermore, a tendency for more enhanced staining for ALP, OC and BSP centrally and Col I, OC and BSP apically was observed in osteoblasts and osteocytes in TCP-P sites compared to TCP-G sites (Table 2, Figure 7a–h). The same was true for Col I staining in the mineralized bone matrix apically (Table 2). In addition, for both test materials higher scores for all markers were recorded centrally compared to apically (Table 2). All sections stained for non-immunized mouse and rabbit IgG remained negative. Taken together, the results of the immunohistochemical analysis showed that with both graft materials bone matrix synthesis and matrix mineralization, and thus bone formation were still actively progressing 6 months after SFA (Figure 7a–h), with a higher activity being present in the central area of the biopsies compared to the apical area. Furthermore, there was a tendency for stronger staining for the osteogenic marker examined in sites grafted with TCP-P compared to TCP-G (Table 2), with significantly higher BSP and Col I expression in osteoblasts (Col I apically) and the osteoid (Col I) in TCP-P sites (Table 2).

### 3.4. Dental Implant Performance 6 Years after Placement

**Figure 10:**
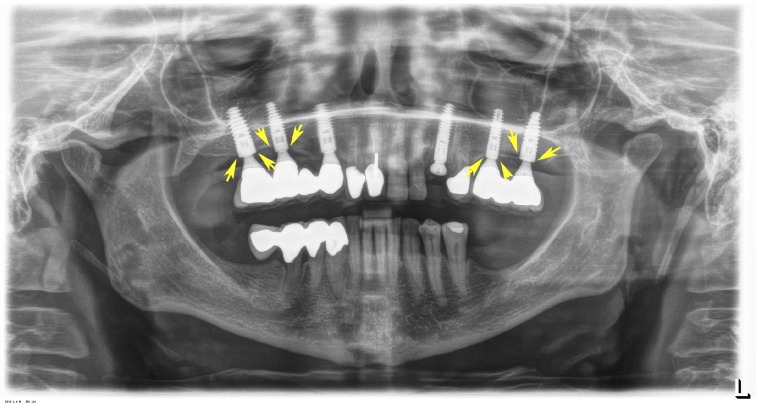
Representative panoramic radiograph showing dental implants without any marginal periimplant bone loss (yellow arrows) 6 years after implant placement in the grafted sinus floors (TCP-P, left; TCP-G, right).

Six years after implant placement the clinical and radiological findings with all implants met the following criteria commensurate with excellent implant success: Clinically, there were no inflammatory changes of the periimplant mucosa visible, and no bleeding upon probing: The depth of the periimplant sulcus was less than 3 mm without any pocket formation being present. Radiologically periimplant bone loss was less than 2 mm. A representative panoramic radiograph is displayed in Figure 10.

## 4. Discussion

The clinical use of β-TCP particulate bone-grafting materials in implant dentistry as well as oral and maxillofacial surgery has been well documented by numerous clinical studies that demonstrated excellent success rates [2,6,8,9,10,11,12]. As a result, tricalcium phosphate is currently regarded as a bone-grafting material well suited for various clinical applications such as SFA procedures. However, clinical studies that directly compare different β-TCP grafting materials with varying material and processing characteristics to each other are still scarce. Since biomaterial science ultimately aims at achieving clinical use, it is of paramount importance to generate knowledge regarding the efficacy of biomaterials in actual patients. In addition, histologic evaluation and histomorphometric assessment are powerful tools for studies on bone repair and regeneration [43]. Accurate histological measurements of the repair process are crucial to validate therapeutic efficacy and address questions about cellular and tissue level responses to biomaterials during repair and regeneration [43]. These measurements can be integrated with radiological, biomechanical and molecular data, thereby providing a comprehensive set of outcome information that corroborate each other. Hence, integration of morphological outcome measurements with radiological data provides significantly more compelling information than either one alone [43]. In clinical studies, dealing with biomaterial stimulated bone repair and regeneration, however, sampling bone biopsy specimens is rarely possible, as most reparative orthopedic procedures do not include a reentry phase, in which removal of newly formed bone tissue for endosseous implant placement is required. As a result, in orthopedics clinical outcomes after biomaterial implantation can only be assessed by less substantive radiological data, standardized clinical pain scales and quality-of-life assessment questionnaires [44,45]. In contrast, in craniofacial surgery bone regenerative procedures exist, in which a staged approach is applied, which features a reentry procedure for dental implant placement into the newly regenerated osseous tissue after biomaterials implantation. Surgical preparation of the implant bed necessitates removal of osseous tissue, which rather than being discarded can be processed for histomorphometric assessment without violating generally accepted ethical standards for patient treatment. In addition, translation of biomaterials to the clinic requires generating knowledge regarding their potency, efficacy and performance in actual patients. Consequently, the objective of the current study was to elucidate the effect of a TCP scaffold material, which was created by combining a HyAc hydrogel matrix with TCP granules, on osteogenesis and bone formation, and to compare the effect of this bone graft material to that of TCP granules of identical chemical composition alone, when used in the same patient, thereby enlarging our knowledge base regarding the behavior of these materials in actual patients in a fundamental way on the basis of histological, histomorphometric and immunohistological findings in human tissues. This was with the goal to develop a TCP-based resorbable bone-grafting scaffold material with advanced surgical handling properties suitable for grafting sites with challenging morphologies and intraoperative conditions as frequently encountered during SFA.

The use of autogenous bone is still considered the gold standard for SFA due to its excellent osteogenic potential [9,12,46,47]. Hence, in the present study 10% (vol %) autogenous bone chips were added to the grafting materials in order to ensure a sufficient supply with osteogenic cells such as osteoblasts and osteoprogenitor cells, which support new bone formation and graft consolidation in combination with the osteoprogenitor cells which migrate from the sinus floor into the grafted area [46,47,48]. Since vascularization and the surface area of the sinus floor, from which osteoprogenitor cells can migrate into the grafted area, are rather limited in the case of SFAs, there is a greater need to add autogenous bone to the bone substitute material compared to other bone defects. Therefore, in the present study the use of autogenous bone was not completely omitted but was limited to a minimal amount, which was added to the bone substitute materials. This way, it became possible to study the effect of TCP-G and TCP-P on bone formation without the results being affected in a major way by the addition of autogenous bone chips, while facilitating biopsy sampling and implant placement as early as 6 months after SFA.

Furthermore, in this study the hypothesis was tested that by using a combination of β-TCP granules and fermented hyaluronic acid, which resulted in a putty-like scaffold, surgical handling properties of the TCP-based grafting material can be enhanced, compared to solely using TCP-granules, without any diminishing effect on the osteoconductive and osteogenic properties of the tricalcium phosphate ceramic. Both TCP test materials had different application characteristics with respect to their clinical use. The putty scaffold had a cohesive moldable structure, while the β-TCP granules displayed a more open structure with retention of the grafting material, however, being more difficult. SFA using both types of TCP grafting material resulted in predictable bone regeneration and degradation of the graft material with formation of sufficient osseous tissue volume for facilitating reliable and stable dental implant placement and anchorage. In the current study a number of advantageous aspects regarding the surgical handling properties were noted for the TCP putty scaffold when compared to the TCP granules. With the putty, applying several layers of the graft material and subsequent condensation were more easily possible. The greater coherence of the putty scaffold resulted in better control for the surgeon when introducing the material into remote areas obstructed from view, while at the same time preventing dislocation of the material due to bleeding and blood flow during surgery, whose extent cannot always be exactly anticipated [49]. In this context, it should be noted that during SFA surgeries there always is the risk of bleeding, which can compromise the surgical outcome due to graft dislocation. Also dislocation of the grafting material into adjacent anatomical regions and migration through the Schneiderian membrane in patients with rather thin Schneiderian membranes can more easily be avoided when using the putty material compared to the use of granules. Consequently, using the TCP putty scaffold may also facilitate grafting sites, in which granules can only be used in combination with membranes and pins for securing the graft material into place. Since it was not possible to measure the application characteristics of the TCP graft materials clinically, evaluation of these application characteristics was only possible in an indirect fashion by assessing the volume stability of the grafted area by analyzing the radiological data. The method presented in the current study for processing the cone-beam CT data sets and determining the graft volume facilitated for the first time visualizing and determining the available bone-volume and its morphology preoperatively in a precise manner. Moreover, the algorithm developed in this study enabled determining the volume of the grafted area thereby reflecting the anatomical situation present clinically in a reproducible manner. There are a number of in vitro methods available for such volumetric measurements, all of which have a number of drawbacks in that they do not adequately take into account postoperative variability such as tissue responses of the Schneiderian membrane, air entrapment in the grafting material and others [50].

Regarding the reduction in graft volume considerable differences were noted between TCP-P and TCP-G in the same patient, which is of great clinical importance in view of being able to insert dental implants of sufficient length. Furthermore, in our study a trend towards lower reduction in graft volume was noticed for the TCP-P material 6 months after SFA, with the difference, however not being statistically significant. It may be speculated that based on the trend a larger powered study including a larger number of patients would suggest that TCP-P may facilitate a lower decrease and thereby a greater stability in grafting volume. The reduction in volume of the grafted area is the result of a number of processes and factors. During early wound healing a condensation of the granules of grafting material may take place. First, there is formation of a blood clot, which is then followed by tissue formation, not-yet mineralized osteogenic mesenchyme, which subsequently becomes fully mineralized woven bone tissue. In parallel, there is increasing gradual resorption of the grafting material and replacement by newly formed bone tissue. From 2 to 3 months after implantation on, woven bone is then remodeled into the cancellous bone displaying the typical microarchitecture of the posterior region of the maxilla, i.e., a combination of bony trabeculae and marrow spaces. Collectively, all these processes lead to a reduction in volume of the grafted area during the 6 months healing period. Due to the involved radiation exposure and ethical considerations, it unfortunately was only possible to acquire a cone-beam CT and thereby calculate the grafting volume at a single time point, i.e., 6 months after SFA. As a result, it is not possible to differentiate between early shrinkage of the graft volume, which occurs within the first postoperative days and secondary shrinkage occurring in the context of bone regeneration and graft resorption. It may be hypothesized that the smaller grain size of the TCP particles used in the putty material may have a beneficial effect on graft shrinkage due to these particles being packed in a more dense fashion after introducing the graft material into the sinus floor [36]. Also the bond between the hyaluronic acid and the TCP particles and the resulting increase in viscoelasticity may contribute to reducing shrinkage during the period, over which resorption of the hyaluronic acid takes place, which can vary from days to weeks [16,17,51,52,53,54]. Which of these factors may have a greater impact on preventing graft shrinkage needs to be elucidated in future studies.

Histologic and histomorphometric analysis showed excellent new bone formation and bone bonding behavior for both TCP-based grafting materials with bone matrix synthesis and matrix mineralization, and thus bone formation, still actively progressing 6 months after SFA. Furthermore, there was significantly greater bone formation and greater osteogenic marker expression, i.e., greater activity with respect to bone matrix formation and maturation for TCP-P. These results are in agreement with findings by Chazono et al. 2004 [13], who implanted TCP-blocks as well as a combination of TCP granules and hyaluronic acid in New Zealand white rabbits and observed slightly greater bone formation in the TCP/hyaluronic acid group. In contrast to our findings, which did not show any osteoclastic resorption of the TCP granules, they, however, noted the presence of osteoclasts. This may be related to the fact that they studied shorter implantation periods. It may be possible that osteoclastic activity is present early on and later on wanes in favor of chemical dissolution mediating the degradation process. Another explanation may be that there is a difference between long bones formed by endochondral ossification and cranial bones, which are formed by intramembranous ossification.

Furthermore, Aslan et al. 2006 [55] demonstrated a stimulatory effect on osteogenesis when solely implanting hyaluronic acid in the rat tibia without additional TCP particles. This is in agreement with cell culture studies that showed a stimulatory effect of HyAc on osteoblast function when using osteoblast derived from neonatal rat calvaria. This effect was both dose-dependent as well as dependent on the molecular weight of the hyaluronic acid used [56].

The results of our study demonstrate that both TCP-G as well as TCP-P supported bone formation and osteoblast differentiation with TCP-P displaying a slightly greater osteogenic potency. This may be related to the physicochemical properties of HyAc, which have been shown to enhance the effect of growth factors [57] as well as the osmotic properties which may lead to a milieu in the grafted area which favors osteoblast proliferation and differentiation [52]. HyAc, itself, however, is not a growth factor, but a polysaccharide, which is a major component of the extracellular matrix. HyAc has FDA approval and a CE mark, thus, use of a putty material which combines TCP granules and 6% recombinant sodium hyaluronate powder should obtain FDA approval more easily than products which combine ceramic bone substitutes with growth factors such as bone morphogenetic proteins or the B2A osteo-inductive growth factor (commercial name AMPLEX). Due to the higher cost involved, growth factor containing bone-grafting materials or tissue engineered products such as the Bioseed^®^ Oral Bone concept have not achieved wide-spread clinical use for SFA at this time [58]. Greater attention to such concepts has been devoted in the context of reconstruction of highly demanding segmental discontinuity defects [58].

Previous histologic and synchroton-CT studies which also facilitated 3D-visualization of biopsies sampled 6 month after SFA with TCP granules of varying porosity showed that bone formation progressed from the sinus floor in an apical direction with the Schneiderian membrane only exhibiting a minor osteogenic potential [2,14]. As a result greater bone formation and particle degradation was noted in the central area compared to the apical area of the biopsies [2]. Consequently, also in the present study immunohistochemical evaluation was performed centrally as well as apically and revealed a considerable gradient with respect to osteogenic marker expression with more enhanced staining for osteogenic markers in the cell and matrix components centrally compared to apically. Furthermore, apically significantly greater staining of osteoblasts for the osteogenic markers Col I and BSP was observed with the TCP-P group compared to TCP-G, which is in excellent correspondence with the histomorphometric results and may support the hypothesis that HyAc may display an additional enhancing effect on osteoblast differentiation compared to TCP alone, which would be of particular importance in the apical region, in which fewer osteogenic cells are present than in the central area with its closer proximity to the native sinus floor. Taken together, the findings of our present study showed that the TCP putty material displayed superior surgical handling properties compared to TCP-G without the HyAc hydrogel matrix having any adverse effect on bone formation, bone tissue maturation or graft volume stability, and thereby confirmed the hypothesis tested.

It also is noteworthy that considerable differences with respect to the amount of bone formed and osteogenic marker expression were detected between individual patients. These findings emphasize the need for examining the effect of age- and gender-related patient individual host factors such as hormone status, body mass index and others on bone regeneration after sinus floor grafting with a given bone-grafting material in additional studies.

## 5. Conclusions

Both TCP-based grafting materials supported bone formation in the augmented sinus floor. Six months after implantation of both TCP materials bone formation and matrix mineralization were still actively progressing in the tissue surrounding the TCP particles. As a result, sinus floor augmentation using both types of TCP grafting materials resulted in favorable bone regeneration and degradation of the graft material with formation of sufficient osseous tissue volume for facilitating stable dental implant placement with all implants being functional without any complications 6 years after implant placement. The TCP putty scaffold material displayed more favorable surgical handling properties compared to TCP-G without the HyAc hydrogel matrix having any adverse effect on bone formation, bone tissue maturation or graft volume stability. The histomorphometric, immunohistochemical and radiologic results showed more enhanced bone formation and osteogenic marker expression and a tendency for greater volume stability for the putty material, however, without the differences in volume stability being statistically significant. Future studies need to clarify whether this effect may be related to the different grain size of the TCP particles used in TCP-P when compared to TCP-G or to the properties of the fermented hyaluronic acid. In conclusion, the results of the current study demonstrate that TCP-P can be regarded as excellent grafting material for SFA in a clinical setting.

## Figures and Tables

**Table 1:** Patient clinical data.

Patient	Age (Years)	Gender	CEROS^®^-TCP-Putty	CEROS^®^-TCP-Granules
1	57	F*	Right	Left
2	63	F	Right	Left
3	70	F	Left	Right
4	72	F	Right	Left
5	66	M	Left	Right
6	67	M*	Left	Right
7	63	F	Right	Left

*–smoker, F—female, M—male.

**Table 2:** Results of the immunohistochemical analysis of osteogenic marker expression in the apical and central areas of biopsies sampled from sites grafted with the TCP-putty scaffold material (TCP-P) or TCP granules (TCP-G).

Col I	Osteoblast		Osteocyte		Fibroblast		Fibrous Matrix of the Osteogenic Mesenchym		Bone Matrix		Osteoid	
**apical**	**TCP-P**	**TCP-G**	**TCP-P**	**TCP-G**	**TCP-P**	**TCP-G**	**TCP-P**	**TCP-G**	**TCP-P**	**TCP-G**	**TCP-P**	**TCP-G**
Mean ± SD	0.85 ± 1	0.14 ± 0.3	0.71 ± 1.4	0.14 ± 0.3	2 ± 1.1	1.71 ± 1.4	1.42 ± 1.2	2.1 ± 1.54	1 ± 1.5	0.4 ± 1.1	1 ± 1.1	0.2 ± 0.75
*p*-value	0.03		0.35		0.6		0.23		0.17		0.03	
**central**	**TCP-P**	**TCP-G**	**TCP-P**	**TCP-G**	**TCP-P**	**TCP-G**	**TCP-P**	**TCP-G**	**TCP-P**	**TCP-G**	**TCP-P**	**TCP-G**
Mean ± SD	1.8 ± 1.4	2.1 ± 1.5	1.4 ± 1.8	0.57 ± 0.5	1.8 ± 2	1 ± 1.4	3.2 ± 1.3	3 ± 0.8	1.2 ± 1.3	1.4 ± 1.5	1 ± 0.5	2.2 ± 1.6
*p*-value	0.64		0.63		0.15		0.36		0.95		0.03	
**ALP apical**	**Osteoblast**		**Osteocyte**		**Fibroblast**		**Fibrous matrix**		**Bone matrix**		**Osteoid**	
	**TCP-P**	**TCP-G**	**TCP-P**	**TCP-G**	**TCP-P**	**TCP-G**	**TCP-P**	**TCP-G**	**TCP-P**	**TCP-G**	**TCP-P**	**TCP-G**
Mean ± SD	1.5 ± 1.7	0.7 ± 1.4	0.14 ± 1.3	0.14 ± 0.3	1.4 ± 1.5	1 ± 1.1	2.8 ± 0.9	2.7 ± 1.9	0.14 ± 0.3	0 ± 0	1 ± 1.4	0.42 ± 0.7
*p*-value	0.18		0.59		0.47		0.65		0.48		0.18	
**central**	**TCP-P**	**TCP-G**	**TCP-P**	**TCP-G**	**TCP-P**	**TCP-G**	**TCP-P**	**TCP-G**	**TCP-P**	**TCP-G**	**TCP-P**	**TCP-G**
Mean ± SD	2.28 ± 1.6	2.1 ± 1.7	1.28 ± 1.6	0.85 ± 1.4	2.4 ± 1.7	2.7 ± 1.7	3.14 ± 0.9	3.8 ± 0.9	0.7 ± 1.1	0.4 ± 0.7	1.5 ± 1.5	1 ± 0.8
*p*-value	0.9		0.25		0.71		0.06		0.47		0.4	
**OC apical**	**Osteoblast**		**Osteocyte**		**Fibroblast**		**Fibrous matrix**		**Bone matrix**		**Osteoid**	
	**TCP-P**	**TCP-G**	**TCP-P**	**TCP-G**	**TCP-P**	**TCP-G**	**TCP-P**	**TCP-G**	**TCP-P**	**TCP-G**	**TCP-P**	**TCP-G**
Mean ± SD	0.85 ± 1.4	0.57 ± 1.5	0.28 ± 0.4	0 ± 0	1.57 ± 1.9	0.57 ± 1.5	2.28 ± 1.6	2.28 ± 1.6	0.14 ± 0.3	0 ± 0	1.28 ± 1.3	0.42 ± 1.1
*p*-value	0.64		0.09		0.2		0.87		0.48		0.04	
**central**	**TCP-P**	**TCP-G**	**TCP-P**	**TCP-G**	**TCP-P**	**TCP-G**	**TCP-P**	**TCP-G**	**TCP-P**	**TCP-G**	**TCP-P**	**TCP-G**
Mean ± SD	2 ± 1.6	1.28 ± 1.6	0.85 ± 1.46	0.14 ± 0.3	1.7 ± 1.7	1.28 ± 1.8	3 ± 1.1	2.7 ± 1.4	0.42 ± 0.5	0.3 ± 0.4	1.14 ± 1	1 ± 0.8
*p*-value	0.3		0.12		0.66		0.52		0.69		0.86	
**BSP apical**	**Osteoblast**		**Osteocyte**		**Fibroblast**		**Fibrous matrix**		**Bone matrix**		**Osteoid**	
	**TCP-P**	**TCP-G**	**TCP-P**	**TCP-G**	**TCP-P**	**TCP-G**	**TCP-P**	**TCP-G**	**TCP-P**	**TCP-G**	**TCP-P**	**TCP-G**
Mean ± SD	1.42 ± 1.8	0.14 ± 0.3	0.57 ± 1.1	0 ± 0	0.57 ± 1.1	0.42 ± 1.1	1.28 ± 1.6	1.28 ± 1.1	0 ± 0	0 ± 0	0.42 ± 0.7	0.28 ± 0.4
*p*-value	0.03		0.09		0.64		0.72		0.99		0.8	
**Central**	**TCP-P**	**TCP-G**	**TCP-P**	**TCP-G**	**TCP-P**	**TCP-G**	**TCP-P**	**TCP-G**	**TCP-P**	**TCP-G**	**TCP-P**	**TCP-G**
Mean ± SD	2.28 ± 1.7	0.85 ± 1	1.14 ± 1.3	0.14 ± 0.3	0.57 ± 1.1	1 ± 1	1.57 ± 1.7	2.7 ± 1.25	0.57 ± 0.5	0.3 ± 0.4	1.14 ± 1	1.2 ± 1.3
*p*-value	0.02		0.018		0.08		0.06		0.25		0.93	
